# Optimisation of scan duration and image quality in oncological ^89^Zr immunoPET imaging using the Biograph Vision PET/CT

**DOI:** 10.1007/s00259-023-06194-4

**Published:** 2023-03-22

**Authors:** Joyce van Sluis, Ronald Boellaard, Rudi A. J. O. Dierckx, Evelien L. M. van Esch, Demi A. Croes, Laura Kist de Ruijter, Pim P. van de Donk, Elisabeth G. E. de Vries, Walter Noordzij, Adrienne H. Brouwers

**Affiliations:** 1 Department of Nuclear Medicine and Molecular Imaging, University Medical Center Groningen, University of Groningen, Hanzeplein 1, 9713 GZ Groningen, The Netherlands; 2 Department of Radiology and Nuclear Medicine, University Medical Centers Amsterdam, Free University of Amsterdam, De Boelelaan 1117, 1081 HV Amsterdam, The Netherlands; 3 Department of Medical Oncology, University Medical Center Groningen, University of Groningen, Hanzeplein 1, 9713 GZ Groningen, The Netherlands

**Keywords:** Zirconium-89, ImmunoPET, SiPM, Image quality, Scan time

## Abstract

**Purpose:**

Monoclonal antibody (mAb)-based PET (immunoPET) imaging can characterise tumour lesions non-invasively. It may be a valuable tool to determine which patients may benefit from treatment with a specific monoclonal antibody (mAb) and evaluate treatment response. For ^89^Zr immunoPET imaging, higher sensitivity of state-of-the art PET/CT systems equipped with silicon photomultiplier (SiPM)-based detector elements may be beneficial as the low positron abundance of ^89^Zr causes a low signal-to-noise level. Moreover, the long physical half-life limits the amount of activity that can be administered to the patients leading to poor image quality even when using long scan durations. Here, we investigated the difference in semiquantitative performance between the PMT-based Biograph mCT, our clinical reference system, and the SiPM-based Biograph Vision PET/CT in ^89^Zr immunoPET imaging. Furthermore, the effects of scan duration reduction using the Vision on semiquantitative imaging parameters and its influence on image quality assessment were evaluated.

**Methods:**

Data were acquired on day 4 post 37 MBq ^89^Zr-labelled mAb injection. Five patients underwent a double scan protocol on both systems. Ten patients were scanned only on the Vision. For PET image reconstruction, three protocols were used, i.e. one camera-dependent protocol and European Association of Nuclear Medicine Research Limited (EARL) standards 1 and 2 compliant protocols. Vision data were acquired in listmode and were reprocessed to obtain images at shorter scan durations. Semiquantitative PET image parameters were derived from tumour lesions and healthy tissues to assess differences between systems and scan durations. Differently reconstructed images obtained using the Vision were visually scored regarding image quality by two nuclear medicine physicians.

**Results:**

When images were reconstructed using 100% acquisition time on both systems following EARL standard 1 compliant reconstruction protocols, results regarding semiquantification were comparable. For Vision data, reconstructed images that conform to EARL1 standards still resulted in comparable semiquantification at shorter scan durations (75% and 50%) regarding 100% acquisition time.

**Conclusion:**

Scan duration of ^89^Zr immunoPET imaging using the Vision can be decreased up to 50% compared with using the mCT while maintaining image quality using the EARL1 compliant reconstruction protocol.

**Supplementary Information:**

The online version contains supplementary material available at 10.1007/s00259-023-06194-4.

## Introduction

The latest generation positron emission tomography (PET) integrated with computed tomography (CT) systems is equipped with silicon photomultiplier (SiPM)-based detector elements. These systems with improved detection capabilities may contribute to enhance diagnostic performance [1–3], but could also allow a reduction in scan duration and/or reduction in administered radioactivity [3, 4].

Over the past decades, antibody-based PET (immunoPET) imaging has become increasingly important in drug development [5]. In addition, it may be a valuable tool to determine which patients may benefit from treatment with a specific monoclonal antibody (mAb) via non-invasive characterisation of tumour lesions, and for evaluation of treatment response [6].

Numerous advantages of zirconium-89 (^89^Zr), such as the long half-life of 78.4 h matching the pharmacokinetic behaviour of antibodies, its relatively low average positron energy of 395 keV resulting in low positron range (3.6 mm in water) for high-resolution PET imaging, and good in vivo stability, make it a suitable candidate for labelling of mAb [7–9]. For ^89^Zr immunoPET imaging, the higher sensitivity of SiPM-based PET/CT systems could be particularly beneficial as the low positron abundance (22.7%) causes the acquired PET images to have a low signal-to-noise level. In addition, the high-energy gamma emission of 909 keV causes high radiation burden and limits the amount of radiotracer that can be administered to patients [10]. Hence, long scan durations are required to obtain adequate statistical image quality, especially at later scan time points.

Efforts to characterise and harmonise ^89^Zr-PET imaging by means of phantom measurement comparisons among different types of systems to ensure quantitative images have been done by Makris et al. [11]. These efforts have been continued by Kaalep et al. [12]. Other system performance comparisons using ^89^Zr-filled phantom studies have been performed by Christian et al. [13] to compare and optimise image quality and phantom sphere (lesion) detectability between systems.

Currently, total body ^89^Zr immunoPET imaging can last up to 2 h acquisition at later time points at 6–7 days postinjection (p.i.), using the conventional photomultiplier tube (PMT)-based mCT Biograph PET/CT (from now on referred to as mCT (Siemens Healthineers)), our clinical reference system. The improved performance characteristics of the SiPM-based Biograph Vision PET/CT (from now on referred to as Vision (Siemens Healthineers)) are expected to lead to improved ^89^Zr immunoPET image quality, and therefore may allow for a reduction in scan duration to increase patient comfort and throughput, and/or administered radioactivity for reduction of radiation exposure.

This study aimed to investigate the difference in semiquantitative performance between the mCT and the Vision PET/CT systems in ^89^Zr immunoPET imaging. In addition, the effects of acquisition time reduction on semiquantitative imaging parameters and its influence on image quality assessment were evaluated.

## Materials and methods

### Patient population

Patients (*n* = 15 (5 men, 10 women; age 33–79, mean ± SD 58 ± 13 years; weight 52–109, mean ± SD 73 ± 15 kg) with cancer with visible [^89^Zr]mAb PET tracer uptake at day 4 p.i. in at least one tumour lesion were enrolled in this prospective study between June 2018 and February 2020 in case of a referral for an ^89^Zr immunoPET acquisition to solve a clinical dilemma [14, 15] or for research purposes (ClinicalTrials.gov identifiers NCT02453984 [16] and NCT04029181 [17]). All patients were scanned on the Vision PET/CT system. Patients (*n* = 5) that underwent the dual acquisition protocol were scanned on both PET/CT systems and gave additional written informed consent after being informed on the study aims, procedures and the additional acquisition of a low-dose CT ($\sim$ 1 mSv). For this purpose, the local medical ethics committee exempted approval without additional procedures (waiver number: METc2017/489).

### Imaging protocol

Patients received an intravenous injection of 37 MBq ^89^Zr-labelled mAb. PET/CT data were acquired on day 4 p.i. On the Vision PET/CT, a standard low-dose CT scan (an X-ray tube current of 43 mAs, a tube voltage of 100 kV and a spiral pitch factor of 1) was performed from the vertex to the toes and used for attenuation and scatter correction. A consecutive emission PET scan was acquired in listmode at 300 s per bed position (s/bp). In case patients were scanned on the mCT, the acquisition parameters of the low-dose CT were as follows: an X-ray tube current of 99 mAs, a tube voltage of 140 kV and a spiral pitch factor of 1.5. PET/CT imaging on the mCT were also performed in listmode at 300 s/bp.

Subsequently, PET listmode data acquired on the Vision were reprocessed to produce additional sets of sinograms corresponding to 225, 150 and 75 s/bp (scan durations are hereinafter referred to as 100%, 75%, 50% and 25% of the acquisition time mimicking shorter scan durations). For reconstruction of Vision PET images, three different protocols were used for each of the four scan durations. We applied the vendor provided reconstruction protocol currently used for [^18^F]FDG imaging, i.e. an ordinary Poisson ordered-subset expectation maximisation (OP-OSEM) 3D-iterative algorithm [18] using 4 iterations, 5 subsets, time-of-flight (ToF) application and resolution modelling, without filtering (hereinafter referred to as the Clinical Vision protocol). In addition, the European Association of Nuclear Medicine (EANM) Research Ltd. (EARL)1 and EARL2 reconstructions [19–21] currently used for quantification of clinically acquired oncological [^18^F]FDG images were obtained using 3D OP-OSEM with 4 iterations and 5 subsets, ToF, with resolution modelling and a Gaussian filter of 7 mm and 5 mm, respectively (hereinafter referred to as the EARL1 and EARL2 Vision protocols). The resulting image size of the images obtained on the Vision was 220 × 220 with a voxel size of 3.3 × 3.3 × 1.5 mm.

For PET data acquired on the mCT, also three different reconstruction protocols were used. The clinically preferred multicentre validated ^89^Zr PET reconstruction protocol [11, 12] using 3D OP-OSEM with 3 iterations, 21 subsets, ToF, resolution modelling and a Gaussian filter of 8 mm (hereinafter referred to as the ^89^Zr-EARL mCT protocol [11]). In addition, images acquired on the mCT were also reconstructed to comply with EARL [^18^F]FDG imaging settings using 3D OP-OSEM with 3 iterations, 21 subsets, ToF, resolution modelling and a Gaussian filter of 6.5 mm (the EARL1 mCT protocol) and a Gaussian filter of 5 mm (the EARL2 mCT protocol). The resulting image size of the images obtained using the mCT was 256 × 256 with a voxel size of 3.2 × 3.2 × 2.0 mm, thus closely matching the image voxel sizes between systems.

### Semiquantitative image analysis

Reconstructed PET/CT data were semiquantitatively analysed using the quAntitative onCology moleCUlaR Analysis suiTE (ACCURATE) version v08072019 [22]. Per image, individual tumour lesions were manually delineated to obtain the maximum and the peak standardised uptake value (SUV_max_ and SUV_peak_) of the tumour. In addition, 1-cm-diameter spherical volumes of interest (VOIs) were placed in healthy tissues: blood pool, kidney cortex and spleen well within the boundaries of the organ to avoid partial volume effects. From these VOIs, SUV_max_, SUV_peak_ and mean standardised uptake value (SUV_mean_) were obtained. A 3-cm-diameter spherical VOI was placed in a homogeneous part of the liver to obtain SUV_max_, SUV_peak_ and SUV_mean_, and to characterise image noise using the standard deviation of the activity within the VOI with regard to the mean activity within the VOI.

### Qualitative image analysis

Images obtained on the Vision at the four different reconstructed scan durations using three different reconstruction protocols were evaluated on image quality. Two nuclear medicine physicians (AHB and WN, with 20 and 5 years of experience in ^89^Zr immunoPET image reading, respectively) independently assessed the images using a dedicated *syngo.*via VB30 (Siemens Healthineers) workstation. All images were scored based on a 5-point Likert scale regarding image noise, lesion margin demarcation and overall image quality (see Supplemental Fig. 1  for the used visual image assessment form).

**Fig. 1:**
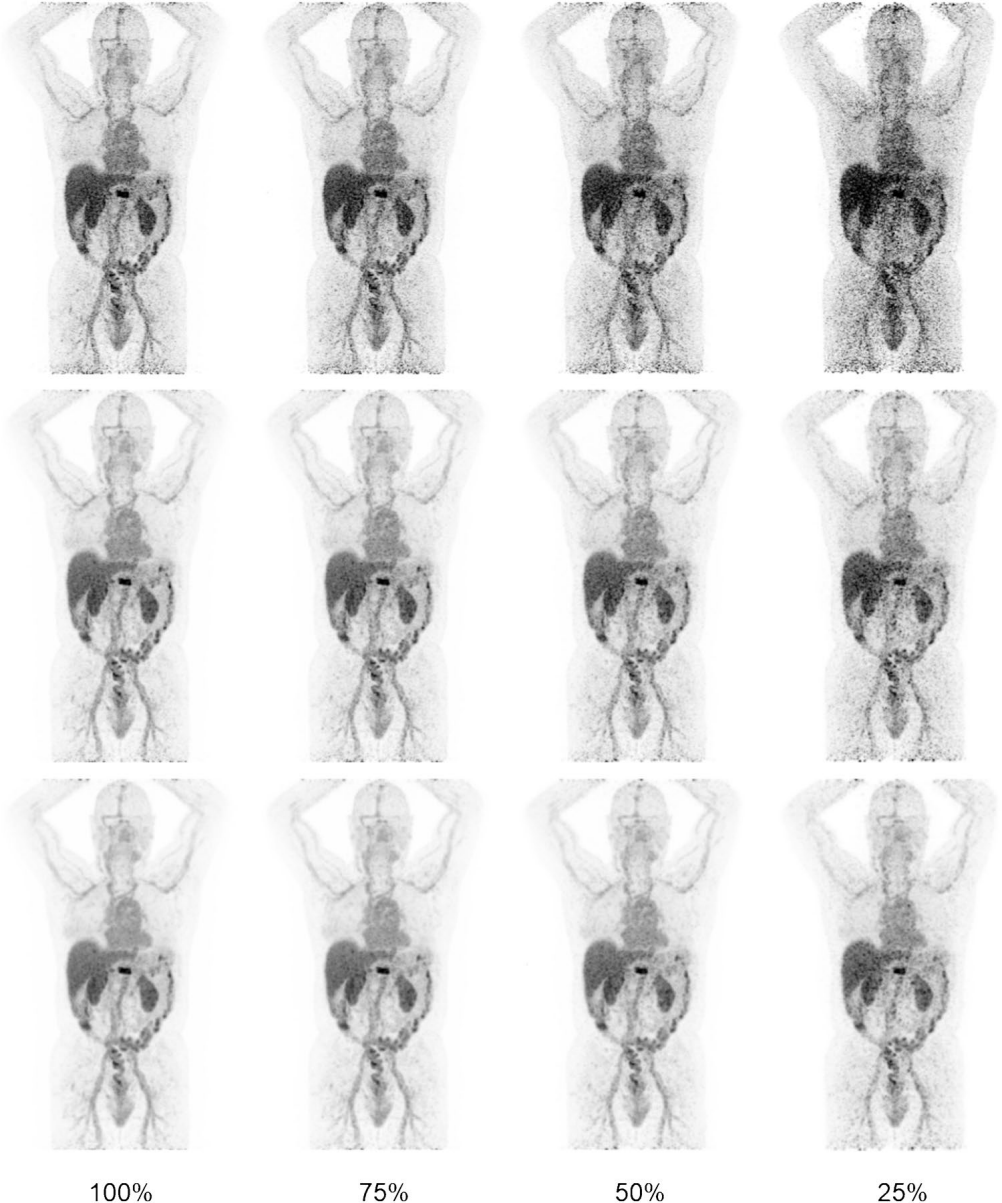
Patient example ^89^Zr immunoPET images obtained using the Vision PET/CT. Maximum intensity projection PET images acquired at day 4 p.i. of 37 MBq [^89^Zr]mAb of a 79-year-old patient (weight 86 kg) with metastatic breast cancer acquired at 100%, 75%, 50% and 25% of the scan duration (from left to right, respectively) using the Clinical Vision, EARL2 Vision and EARL1 Vision reconstruction protocols (from top to bottom, respectively). Images were scaled at equal contrast intensities

### Statistical analysis

Statistical analyses were performed in SPSS Statistics, version 25.0 (IBM Corp.). To evaluate the difference in semiquantitative performance between systems, non-parametric Wilcoxon signed rank tests were conducted. The difference in lesion SUV_max_ and SUV_peak_ and the difference in healthy tissue SUV_max_, SUV_peak_ and SUV_mean_ between systems were explored.

For each reconstruction method performed to obtain images on the Vision, lesion SUV_max_ and SUV_peak_ per scan duration, 75%, 50% and 25%, were compared with the lesion SUV_max_ and SUV_peak_ of images acquired at 100% of the count time. A repeated-measures analysis of variance (ANOVA) with post hoc Bonferroni adjustment for pairwise comparison was used. A *P* value of less than 0.05 was considered significant. This comparison was also performed for assessing the difference in healthy tissue SUV_max_, SUV_peak_ and SUV_mean_ between the 100% scan time images and images acquired at shorter scan durations.

Furthermore, the standard deviation of the voxel values within the liver VOIs was compared between the 100% scan time images and images acquired at shorter scan durations as well.

Inter-reader agreement concerning image noise, tumour lesion demarcation and overall image quality was analysed using kappa statistic. To this aim, the original 5-point scores were reassigned to 2-point scores: 1 + 2 + 3 became 1, and 4 + 5 became 2. A *P* value of less than 0.05 was considered significant.

## Results

### Semiquantitative image analysis

Five patients were scanned on both PET/CT systems and acquired images were evaluated for the semiquantitative performance comparison of the mCT versus Vision. Each acquisition using the mCT resulted in three images (obtained using three different reconstruction protocols), whereas each acquisition using the Vision resulted in 12 images (three reconstruction methods times four scan durations). For illustrative purposes, Fig. 1 shows example patient PET/CT images acquired using the three reconstruction protocols at different scan durations ranging from 100 to 25%. From top to bottom, Fig. 1 shows images obtained using the Clinical Vision, EARL2 Vision and EARL1 Vision reconstruction protocol, arranged by amount of applied smoothing from least to most. In the Clinical Vision images obtained using 100% scan duration, a clearly demarcated lesion in the vertebra is visible; however, the image has a noisy (speckly) outlook which becomes more disturbing towards shorter acquisition times. The images reconstructed according to EARL2 Vision settings show a sharply demarcated lesion and, because of the applied 5-mm Gaussian filter, the noise is smoothed away which prevents the speckly noise appearance to take the upper hand, also in the images obtained with shorter scan durations. The EARL1 Vision reconstructed images still clearly show the lesion in the vertebra, however apart from smoothing away the noise, the 7 mm Gaussian filter also smooths the edges of the lesion causing a slightly blurred demarcation. Regarding shorter scan durations, the EARL1 reconstructed images show the highest robustness to noise with the least increase in speckly noise pattern towards shorter acquisition times.

For each of the five patients scanned on both the mCT and Vision, a total of 15 images were obtained, whereas 12 images were obtained for each of the 10 patients undergoing acquisition on the Vision only. Overall, 195 images were collected and tumour segmentations were performed on each of the images individually. A total of 5 tumour lesions were found in the double scans, a single lesion per patient. The lesions were first identified on the images acquired on the mCT, our clinical reference system, and subsequently confirmed on the Vision images. Furthermore, 17 tumour lesions were included in the single acquisitions obtained using the Vision PET/CT (a total of 2 tumour lesions per tissue type per patient). In total, 279 tumour segmentations were made (a segmentation for each reconstruction method and acquisition time resulted for the acquisitions on the mCT in three segmentations per lesion (i.e. 5*3 tumour segmentations), and for the Vision in 12 segmentations per lesion (i.e. (17 + 5)*12 tumour segmentations).

Concerning the double acquired scans, median lesion SUV_max_ and SUV_peak_ derived from the images obtained using the mCT and the EARL standard 1 compliant harmonised reconstruction protocol were 14.1 (range 2.4–35.7) and 10.3 (range 1.7–14.4). For the dual images obtained with the Vision, median lesion SUV_max_ and SUV_peak_ derived from images reconstructed according to the EARL1 standard compliant protocol were 17.9 (range 4.1–29.7) and 10.1 (range 2.0–13.6), respectively. An overview of median lesion SUV_max_ and SUV_peak_ comparison between PET/CT systems obtained using different reconstruction protocols can be found in Table 1. Regarding the included healthy tissues, a comparison of semiquantitative parameters between PET/CT systems is shown in Table 2.

**Table 1:** Tumour lesion median SUV_max_ and SUV_peak_ comparison between both systems (only shown for acquisitions on both PET/CT systems (*n* = 5)

Tissue		Biograph mCT			Biograph Vision	
	SUV_max_*median (range)*		SUV_peak_ *median (range)*	SUV_max_ *median (range)*		SUV_peak_ *median (range)*
Tumour lesions *Reconstruction protocol:*						
^89^Zr-EARL/clinical	12.7 (2.2–27.4)		9.8 (1.8–11.9)	25.6 (7.0–64.1)		11.9 (2.1–14.7)
EARL2	15.5 (2.8–48.3)		10.9 (1.8–18.2)	17.9 (4.1–29.7)		10.7 (1.9–13.7)
EARL1	14.1 (2.4–35.7)		10.3 (1.7–14.4)	15.7 (3.4–22.0)		10.1 (2.0–13.6)

**Table 2:** Healthy tissue median SUV_max_, SUV_peak_ and SUV_mean_ comparison between systems (only shown for acquisitions on both PET/CT systems (*n* = 5))

Tissue	Biograph mCT			Biograph Vision		
	SUV max *median (range)*	SUV peak *median (range)*	SUV mean *median (range)*	SUV max *median (range)*	SUV peak *median (range)*	SUV mean *median (range)*
Blood pool *Reconstruction protocol:*						
^89^Zr-EARL/clinical	10.7 (4.3–13.7)	7.3 (3.7–10.5)	9.11 (3.7–9.8)	14.7 (11.1–25.4)	7.8 (6.3–12.2)	8.6 (6.9–15.0)
EARL2	12.8 (7.0–18.0)	7.4 (5.3–12.1)	9.7 (5.6–11.4)	9.7 (8.5–18.9)	7.2 (5.8–11.7)	8.2 (6.4–14.6)
EARL1	11.9 (6.6–13.8)	7.1 (5.1–11.7)	9.5 (5.5–12.0)	9.0 (7.1–16.4)	7.0 (5.5–11.4)	7.9 (6.4–14.1)
Kidney cortex *Reconstruction protocol:*						
^89^Zr-EARL/clinical	5.9 (3.1–8.7)	5.8 (3.1–6.5)	5.6 (3–6.9)	5.9 (6.6–19.1)	6.9 (4.0–7.8)	6.3 (4.2–9.3)
EARL2	7.6 (3.6–9.7)	5.3 (3.0–6.2)	5.0 (3.1–7.9)	7.8 (5.9–10.2)	6.9 (3.8–8.1)	6.4 (3.9–8.3)
EARL1	6.9 (4.9–9.0)	5.7 (3.9–6.5)	5.8 (3.3–7.7)	7.4 (5.4–13.9)	6.7 (3.7–8.1)	6.4 (3.8–7.7)
Spleen *Reconstruction protocol:*						
^89^Zr-EARL/clinical	4.3 (3.6–7.6)	3.6 (3.3–7.5)	3.2 (2.6–7.4)	7.7 (6.6–17.3)	4.3 (4.0–8.6)	4.6 (1.9–9.3)
EARL2	5.2 (2.1–7.8)	3.6 (2.1–7.1)	3.6 (1.8–5.6)	6.0 (4.3–11.5)	4.7 (3.5–8.4)	5.0 (2.4–9.1)
EARL1	4.4 (3.4–7.6)	3.6 (3.2–7.0)	3.3 (3.11–6.0)	5.5 (3.7–10.5)	4.7 (3.4–8.2)	3.3 (3.1–5.0)
Liver *Reconstruction protocol:*						
^89^Zr-EARL/clinical	8.6 (8.1–9.8)	6.8 (6.4–8.1)	4.9 (4.3–6.0)	11.9 (10.3–23.0)	6.0 (4.8–8.1)	5.4 (4.0–6.1)
EARL2	11.9 (10.2–14.0)	6.8 (6.1–8.7)	5.2 (4.3–6.2)	9.0 (6.5–13.4)	6.1 (4.7–7.9)	5.3 (4.0–6.1)
EARL1	9.5 (8.2–11.6)	7.0 (6.6–8.8)	4.9 (4.3–6.4)	8.4 (5.7–11.4)	6.0 (4.9–7.8)	5.3 (4.0–6.2)

**Fig. 2:**
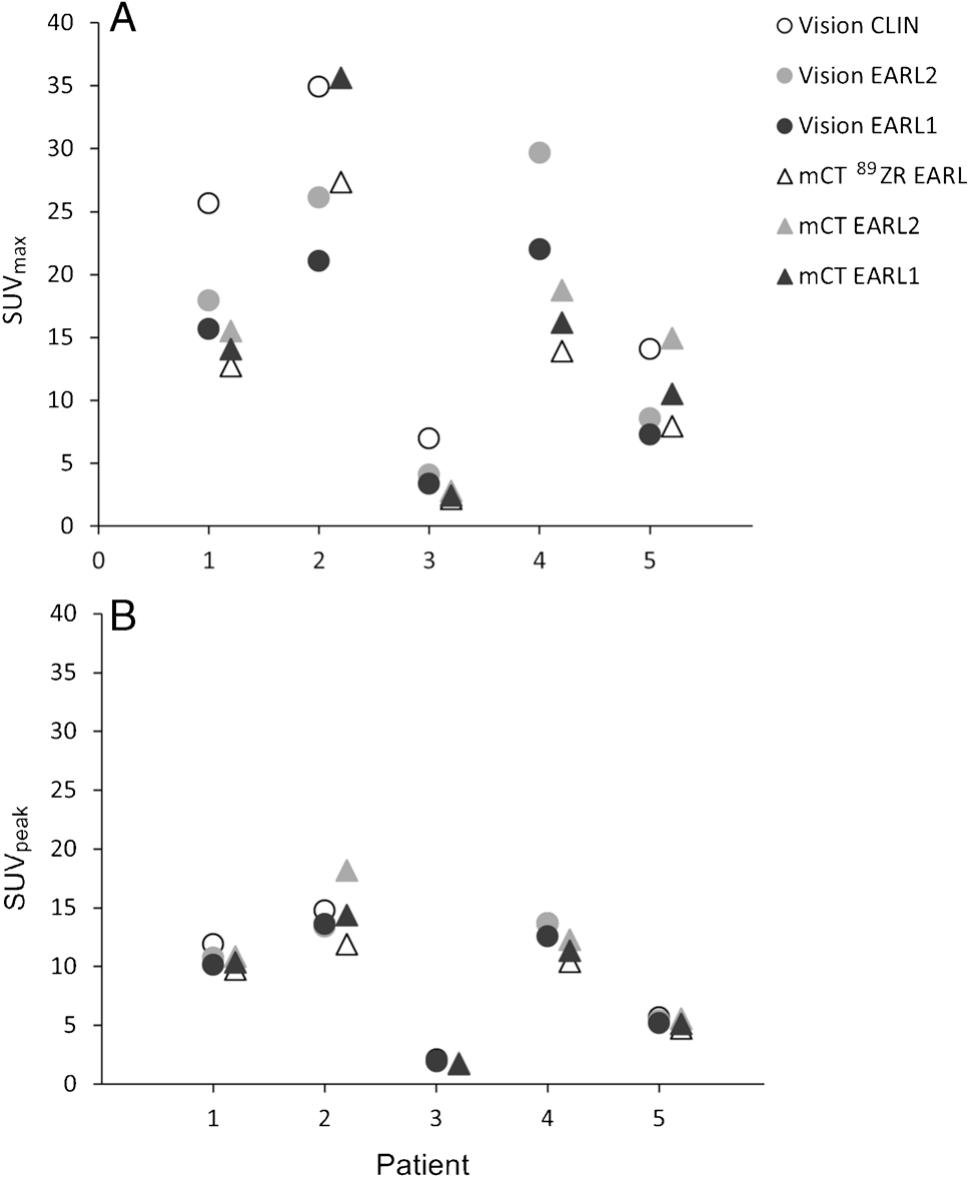
Semiquantitative tumour lesion comparison between PET/CT systems. For each patient (*n* = 5), the lesion SUV_max_ (**A**) and SUV_peak_ (**B**) derived from images obtained with the Vision PET/CT system and using the Clinical Vision (Vision CLIN) (white dot), the EARL2 Vision (grey dot) and the EARL1 Vision reconstruction protocol (black dot) are compared directly with tumour lesion SUVs derived from images using the mCT PET/CT system and ^89^Zr-EARL mCT, EARL2 mCT and EARL1 mCT reconstruction protocols (white, grey and black triangles, respectively). Please note, for readability reasons, the tumour lesion SUV_max_ outliers found in patient 4 of 64.1 obtained using the Clinical Vision protocol and of patient 2 of 48.3 obtained using the EARL2 mCT protocol are not shown in subfigure **A**

**Fig. 3:**
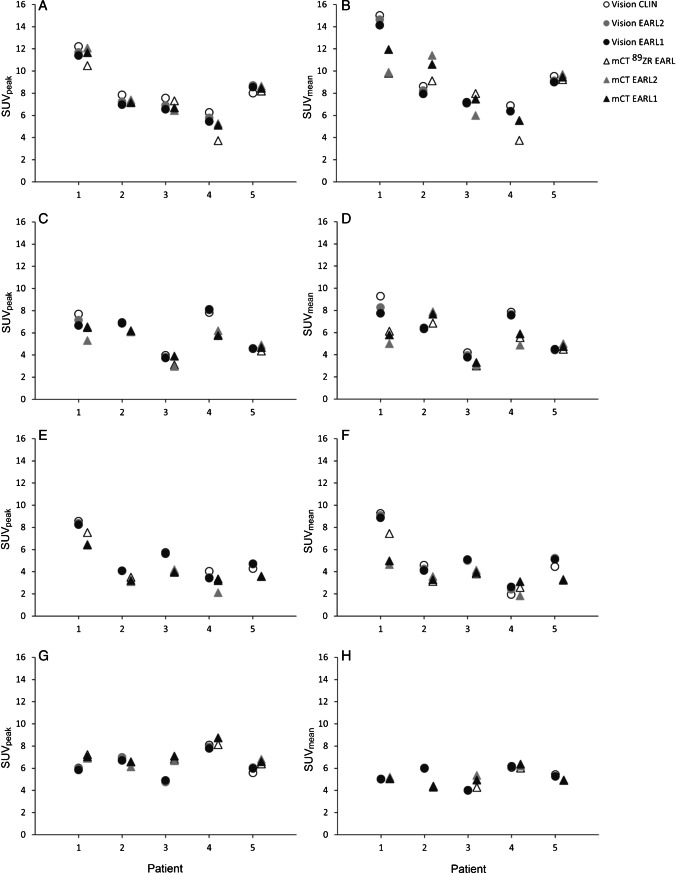
Semiquantitative healthy tissue compared between PET/CT systems. For each patient (*n* = 5), SUV_peak_ (left column) and SUV_mean_ (right column) of healthy tissues (blood pool (**A** and **B**), kidney cortex (**C** and **D**), spleen (**E** and **F**) and liver (**G** and **H**)) derived from images obtained through use of the Vision PET/CT system and the Clinical Vision (Vision CLIN) reconstruction protocol (white dot), the EARL2 Vision reconstruction protocol (grey dot) and the EARL1 Vision reconstruction protocol (black dot) are compared directly with healthy tissue SUVs derived from images using the mCT PET/CT system and.^89^Zr-EARL mCT, EARL2 mCT and EARL1 mCT reconstruction protocols (white, grey and black triangles, respectively)

Scatter plots in Fig. 2 show the difference in lesion SUV_max_ and SUV_peak_ between images derived from both PET/CT systems. For each of the healthy tissues, a similar comparison between systems was performed using SUV_max_, SUV_peak_ and SUV_mean_ (see Fig. 3). The results from the semiquantitative performance comparison between PET/CT systems using Wilcoxon signed rank tests are shown in Table 3; no significant difference is indicated with ‘equivalent’. A significant difference in lesion SUV_max_ and SUV_peak_ was found between systems when comparing the images obtained using the Clinical Vision protocol and the ^89^Zr-EARL mCT protocol (*Z* =  − 2.02, *P* < 0.05); no significant differences in tumour lesion SUV_max_ and SUV_peak_ were found when comparing system semiquantitative performance using the EARL standard compliant settings 1 and 2. Concerning the healthy tissues, significantly different SUV_max_ were found between systems in the blood pool, kidney cortex, spleen and liver when using the Clinical Vision and ^89^Zr-EARL mCT reconstruction protocol (*P* < 0.05). Using these reconstruction settings, SUV_peak_ measured in the kidney cortex differed significantly between systems as well (*Z* =  − 2.02, *P* < 0.05). No significant differences in healthy tissue SUV_max_, SUV_peak_ and SUV_mean_ were found when comparing system semiquantitative performance using the EARL standard compliant settings 1 and 2 for the blood pool, kidney cortex and liver. However, for the spleen, significant differences between systems regardless of reconstruction settings and semiquantitative parameters were observed (*P* < 0.05). Table 2 shows an overall increase of approximately 25% in median SUV for the spleen irrespective of reconstruction protocol.

**Table 3:** Semiquantitative performance in tumour lesion comparison between both PET/CT systems (*n* = 5) using the Wilcoxon signed rank test

	*Z*	*P* value	Equivalence
**Tumour lesions** *Reconstruction protocol:*			
^89^Zr-EARL/clinical			
SUV_max_	− 2.02	0.04	No
SUV_peak_	− 2.02	0.04	No
EARL2			
SUV_max_	− 0.14	0.89	Yes
SUV_peak_	− 0.67	0.50	Yes
EARL1			
SUV_max_	− 0.14	0.89	Yes
SUV_peak_	− 0.41	0.69	Yes

**Fig. 4:**
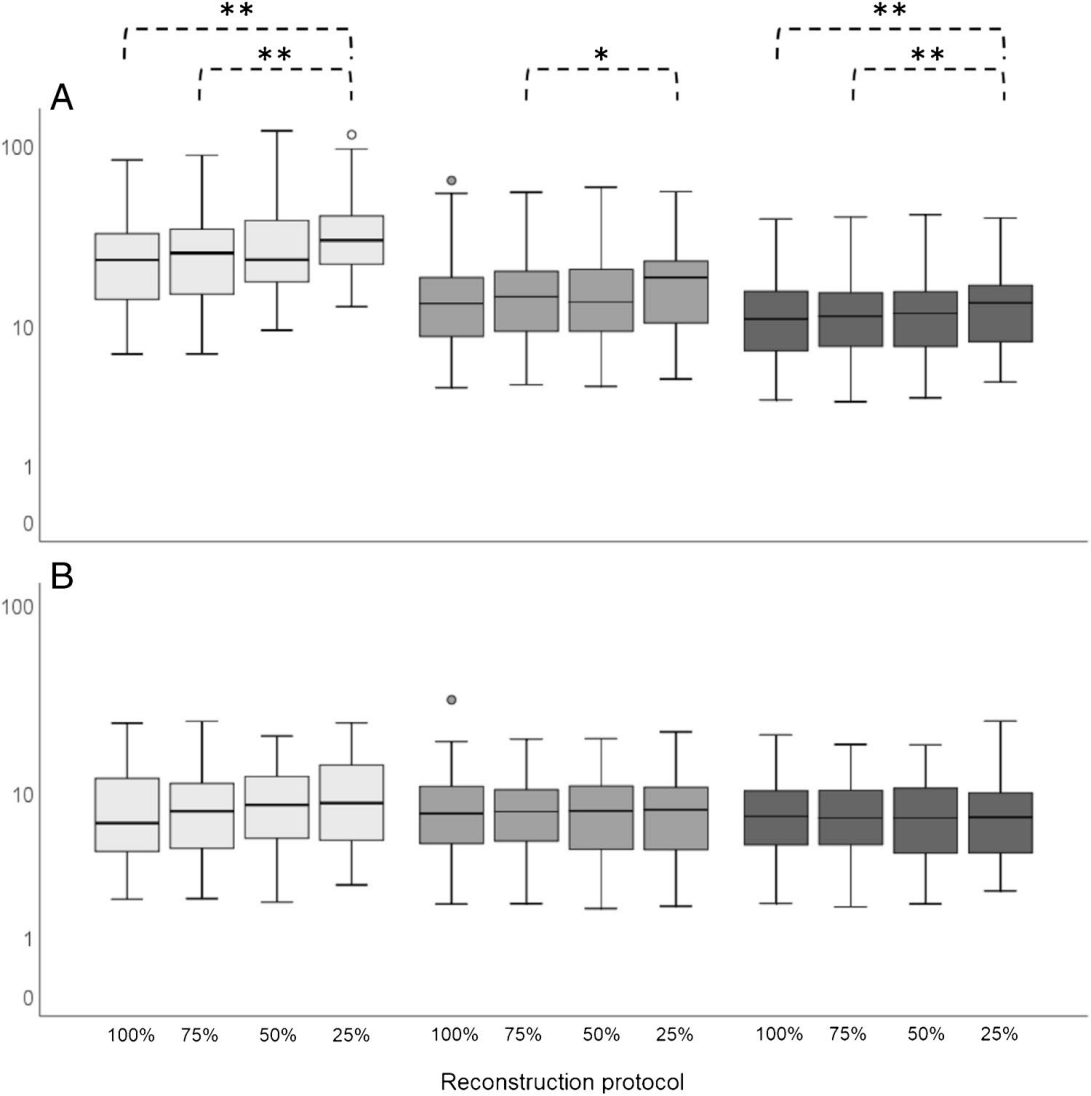
Vision only semiquantitative tumour lesion comparison. For all patients (*n* = 15), the tumour lesion SUV_max_ (**A**) and SUV_peak_ (**B**) derived from images obtained using the Vision PET/CT system and the Clinical Vision reconstruction protocol (light grey), the EARL2 Vision reconstruction protocol (grey) and the EARL1 Vision reconstruction protocol (dark grey) are compared at different scan durations (100 to 25%, from left to right for each reconstruction protocol). The boxes bound the interquartile range (IQR) divided by the median SUV. The whiskers extend to a maximum of 1.5*IQR beyond the box. $$*$$ indicates *P* < 0.05 and $$**$$ indicates *P* < 0.01. Outliers are represented by dots

Boxplots in Fig. 4 show lesion SUV_max_ and SUV_peak_ comparisons between different scan durations derived from images obtained using the Vision. A significant difference was found in lesion SUV_max_ between images using the Clinical Vision reconstruction protocol obtained at 100% scan duration and 25% (*P* = 0.009, 95% CI (− 18.0 to − 2.0)), and between images obtained at 75% scan duration and 25% (*P* = 0.008, 95% CI (− 17.1 to − 2.0)). When using the EARL2 Vision reconstruction settings, lesion SUV_max_ differed significantly between 75 and 25% scan duration (*P* = 0.02, 95% CI (− 5.5 to − 0.3)). Images reconstructed using the EARL1 Vision protocol showed significant differences in lesion SUV_max_ between 100 and 25% scan duration (*P* = 0.001, 95% CI (− 2.3 to − 0.5)), and between 75 and 25% (*P* = 0.009, 95% CI (− 2.5 to − 0.3)). No significant differences were found in lesion SUV_peak_ between images obtained at different scan durations.

**Fig. 5:**
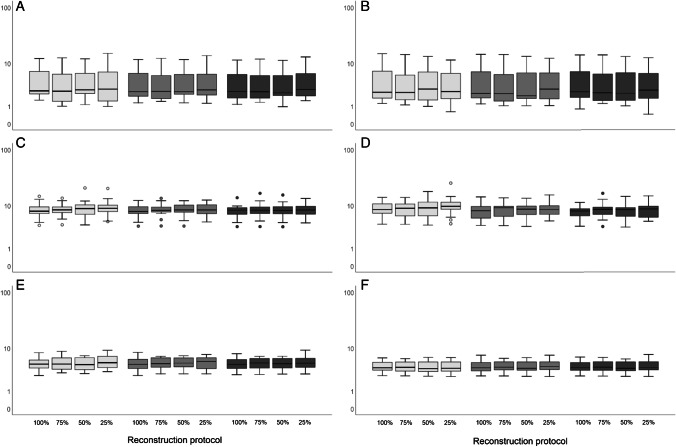
Vision only semiquantitative healthy tissue comparison between scan durations. For all patients (*n* = 15), SUV_peak_ (left column) and SUV_mean_ (right column) of healthy tissues (blood pool (**A** and **B**), kidney cortex (**C** and **D**), spleen (**E** and **F**) and liver (**G** and **H**)) derived from images obtained through use of the Vision PET/CT system and the Clinical Vision reconstruction protocol (light grey), the EARL2 Vision reconstruction protocol (grey) and the EARL1 Vision reconstruction protocol (dark grey) are compared at different scan durations (100 to 25%, from left to right for each reconstruction protocol)

The results of the healthy tissue comparisons between scan durations are shown in Fig. 5. No significant differences in healthy tissue SUV_mean_ and SUV_peak_ were found between images obtained using the Vision at different scan durations. Because of different ^89^Zr-labelled mAbs used in this study, a substantial visual difference was observed in the uptake in the spleen. For clarity, the healthy tissue comparisons per ^89^Zr-labelled mAb for the spleen are shown in Supplemental Fig. 2 .

**Fig. 6:**
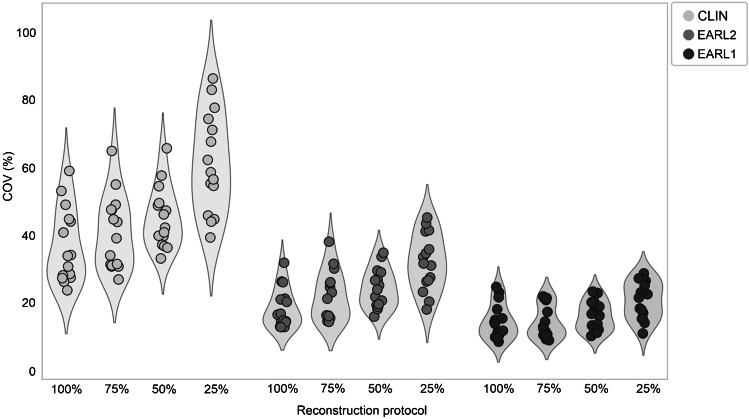
Liver COV (*n* = 15) obtained from the Clinical Vision (light grey), EARL2 Vision (grey) and EARL1 Vision compliant (dark grey) reconstructed images at 100%, 75%, 50% and 25% of the scan time (from left to right)

Noise estimates from calculation of the coefficient of variation (COV) derived from the 3-cm-diameter liver VOIs in each image are shown in Fig. 6. A difference in image noise levels can be observed between reconstruction methods and scan durations. Noise levels increase with shorter scan times; this effect is more prominent when using the Clinical Vision reconstruction protocol compared to EARL2 and EARL1 compliant reconstruction settings.

### Qualitative image analysis

Vision only images (*n* = 15 patients) were reconstructed using three different reconstruction protocols and visually assessed on noise levels, lesion demarcation and overall image quality. Highest mean scores on noise levels and overall image quality were assigned to the images reconstructed according to the EARL1 Vision protocol (mean scores on noise and image quality of 4.3 and 4.4 at 100% of the scan duration, 3.6 and 3.9 at 75% and 3.1 and 3.3 at 50%, respectively). The EARL2 Vision reconstructed images received a slightly higher appreciation on lesion demarcation with respect to the EARL1 Vision reconstructed images (mean score of 4.4 versus 4.2 at 100% of the scan duration, 4.1 versus 3.9 at 75% and 3.7 versus 3.5 at 50%).

Inter-reader agreement ranged from fair to moderate on noise, lesion demarcation and overall image quality with $\kappa$ = 0.23 (*P* = 0.000, 95% CI (0.12–0.34)), $\kappa$ = 0.27 (*P* = 0.000, 95% CI (0.13–0.41)) and $\kappa$ = 0.41 (*P* = 0.000, 95% CI (0.28–0.55)), respectively.

## Discussion

In the current study, semiquantitative performance of the Vision with regard to its predecessor, the mCT, was evaluated for ^89^Zr immunoPET imaging in oncology patients. In addition, possibilities of reducing scan time while maintaining image quality using the Vision were explored.

This study shows that when using the Vision PET/CT system, a reduction in scan time of 50% is possible regardless of reconstruction settings according to Fig. 4. However, the use of images reconstructed according to EARL2 Vision or the Clinical Vision protocol would result in too much elevated noise levels (see Fig. 6). Therefore, we recommend to apply the EARL1 Vision settings for image reconstruction for a 50% reduction in scan duration while maintaining semiquantitative PET image accuracy.

Significant differences in semiquantitative PET image parameters were found for shorter scan durations when using the Clinical Vision reconstruction protocol, especially SUV_max_ increases at shorter scan times (50% and 25%) with regard to 100% acquisition time. A non-negligible consideration when using SUV_max_ for uptake measurements in PET images is statistical quality. When reducing scan time, variability in SUV_max_ can largely be explained by the associated increase in noise [23] (see Fig. 6). The addition of a Gaussian filter (as used in the EARL2 and EARL1 Vision reconstruction settings (7 mm and 5 mm, respectively)) smooths the image hereby reducing noise and SUV_max_ variability [24] which results in similar results for lesion quantification at shorter scan times.

A more robust alternative, and diminishing the need for a filter at shorter acquisition times, is to use SUV_peak_ for lesion quantification. Although SUV_peak_ is expected to be more susceptible to partial volume effect in small lesion segmentation (19), its semiquantitative performance is less affected by scan duration and reconstruction protocol (see Figs. 4 and 5). Makris et al. [11] previously recommended the use of SUV_peak_ for performing semiquantitatively accurate ^89^Zr immunoPET imaging studies. They found very low variability in SUV_peak_ between various PET/CT systems and imaging sites. Moreover, this recommendation has then been affirmed in an ^89^Zr immunoPET imaging study by Kaalep et al. [12] describing a multicentre PET/CT system and reconstruction comparison trial in which SUV_peak_ was found to be least sensitive to noise and reconstruction differences. As we also found SUV_max_ to vary not only between reconstructed images obtained from different PET/CT systems, but also between differently reconstructed images obtained from a single system, our recommendation is (in line with previously reported results described above) to use SUV_peak_ for quantification of ^89^Zr immunoPET images. In case EARL standard 1 compliant settings are used (for comparison between systems), SUV_max_ could be reported as well for lesion quantification besides SUV_peak_.

In order to obtain quantitatively comparable results, we standardise and harmonise PET imaging procedures [19] incorporating various methods, including different amounts of applied smoothing. With the introduction of new PET systems with improved performance characteristics, the harmonisation and standardisation specifications are updated as well [20] to preserve some of the improved image quality that can be obtained. However, in the case of ^89^Zr immunoPET imaging, only a low amount of activity can be administered (37 MBq) due to the long half-life associated with high radiation exposure. This low amount of activity in addition to the low positron abundance causes image quality of ^89^Zr immunoPET images to be in the bottom range; low count statistics result in overall poor image quality. Hence, reconstruction protocols that smooth in excess, such as incorporated in the EARL1 compliant reconstruction settings, are still required under these circumstances.

In the current study, the difference between SUV_peak_ and SUV_mean_ derived from healthy tissues is minimal due to the small 1-cm-diameter spherical VOI that was used in the blood pool, kidney cortex and spleen. SUV_mean_ was included in the evaluation of semiquantitative performance comparison of healthy tissues between systems as SUV_mean_ measurement of these organs is used, for example, in whole organ dosimetry analyses. Using SUV_mean_ for whole organ dosimetry avoids susceptibility to segmentation variability as opposed to using SUV_max_ or SUV_peak_ [25]. Please note, the significant difference in SUV_max_ for tumour lesions and all healthy tissues found between systems using the Clinical Vision reconstruction protocol versus the ^89^Zr-EARL mCT protocol (see Tables 3 and 4). This difference can be explained by the 8-mm Gaussian smoothing filter applied to the images acquired using the mCT, whereas images acquired on the Vision were not smoothed at all. With regard to semiquantitative performance between systems at 100% acquisition time, no further significant differences between tumour lesions and blood pool, kidney cortex and liver measurements were found. For the spleen however, a significant increase in SUV measured on the images obtained from the Vision PET/CT was observed with respect to the images obtained from the mCT PET/CT. This was only the case for the spleen (and not for the other healthy tissues). Thus far, we have not found a plausible explanation. We suspect the improved tissue demarcation due to improved sensitivity and ToF on the Vision PET/CT system could play a role here. No patient instructions with respect to food and fluid intake prior to ^89^Zr immunoPET acquisition were given, resulting in large cold areas with the size of a filled stomach on the acquired images. These cold spots were more prominently visible on the Vision images with a clearer demarcation of the stomach. Due to the improved ToF, better contrast recovery in the spleen using the Vision PET/CT may have resulted in a better reflection of the true counts originating from the spleen as opposed to some possible larger signal spill over between the cold stomach and the very intense spleen on the mCT images. Future ^89^Zr immunoPET studies with a larger homogeneous patient population will have to explore this phenomenon to clarify these findings. Until then, the spleen should not be used as reference tissue in ^89^Zr immunoPET imaging studies.

**Table 4:** Quantitative performance in healthy tissue comparison between systems (*n* = 5) using the Wilcoxon signed rank test

	*Z*		*P*value	Equivalence
**Healthy tissues** Blood pool *Reconstruction protocol:* ^89^Zr-EARL/clinical				
SUV_max_		− 2.02	0.04	No
SUV_peak_		− 1.75	0.08	Yes
SUV_mean_		− 0.67	0.50	Yes
EARL2				
SUV_max_		− 0.14	0.89	Yes
SUV_peak_		− 0.67	0.50	Yes
SUV_mean_		− 0.67	0.50	Yes
EARL1				
SUV_max_		− 0.67	0.50	Yes
SUV_peak_		− 0.41	0.69	Yes
SUV_mean_		− 0.14	0.89	Yes
Kidney cortex *Reconstruction protocol:* ^89^Zr-EARL/clinical				
SUV_max_		− 2.02	0.04	No
SUV_peak_		− 2.02	0.04	No
SUV_mean_		− 1.21	0.23	Yes
EARL2				
SUV_max_		− 1.21	0.23	Yes
SUV_peak_		− 1.75	0.08	Yes
SUV_mean_		− 0.94	0.35	Yes
EARL1				
SUV_max_		− 1.21	0.23	Yes
SUV_peak_		− 0.94	0.35	Yes
SUV_mean_		− 0.94	0.35	Yes
Spleen *Reconstruction protocol:* ^89^Zr-EARL/clinical				
SUV_max_		− 2.02	0.04	No
SUV_peak_		− 2.02	0.04	No
SUV_mean_		− 1.75	0.08	Yes
EARL2				
SUV_max_		− 1.48	0.14	Yes
SUV_peak_		− 2.02	0.04	No
SUV_mean_		− 2.02	0.04	No
EARL1				
SUV_max_		− 2.02	0.04	No
SUV_peak_		− 2.02	0.04	No
SUV_mean_		− 1.75	0.08	Yes
**Liver**				
*Reconstruction protocol:*				
SUV_max_		− 2.02	0.04	No
SUV_peak_		− 1.48	0.14	Yes
SUV_mean_		− 0.67	0.50	Yes
EARL2				
SUV_max_		− 1.21	0.23	Yes
SUV_peak_		− 1.21	0.23	Yes
SUV_mean_		− 0.14	0.89	Yes
EARL1				
SUV_max_		− 0.94	0.35	Yes
SUV_peak_		− 1.75	0.08	Yes
SUV_mean_		− 0.14	0.89	Yes

Regarding qualitative image assessment, overall, the images reconstructed according to the EARL1 protocol received the highest scores resulting from the visual assessment; higher mean scores were obtained for noise levels and overall image quality, and there was only a slight difference in lesion demarcation scores in favour of the EARL2 reconstructed images. Furthermore, fair to moderate inter-reader agreement was achieved. As the nuclear medicine physicians were asked to score the images individually without a direct comparison with optimal ^89^Zr immunoPET image quality, scoring was considered difficult. Another factor that should be considered when interpreting these results is that the image quality of [^18^F]FDG PET/CT scans (compared to ^89^Zr PET/CT images) on the Vision is excellent. These excellent [^18^F]FDG PET images might have been an unintentional reference for visual ^89^Zr immunoPET image assessment. Furthermore, previous experience with ^89^Zr immunoPET readings, and personal preference of the reading Nuclear Medicine physicians regarding acceptable image quality could have played a role in the observed variation in the image quality assessment.

Previous work also performed by our research group explored the effect of scan time reduction on semiquantitative PET image parameters and image quality in [^18^F]FDG PET imaging using the Vision [4]. Here, a factor 3 reduction in scan time was considered possible while maintaining image quality using the clinically preferred Vision reconstruction protocol with additional 2-mm Gaussian filtering. In the current study, possibilities to reduce scan duration in ^89^Zr immunoPET imaging were explored. Using the EARL Vision protocols, semiquantitative performance remains reliable when decreasing scan duration up to a factor of 2 (see Figs. 4 and 5) at the cost of a slight increase in noise (see Fig. 6). Therefore, for ^89^Zr immunoPET imaging in the clinic using an SiPM-based PET/CT, one may choose to reduce scan duration to improve patient comfort and increase throughput. On the other hand, in case of paediatric patients or for non-life-threatening diseases, an equally proportional reduction in the amount of injected activity would be recommended to reduce radiation exposure. However, as shown before for [^18^F]FDG PET studies [4], more room for optimising image quality by changing amount of administered activity and/or scan duration is feasible because of the higher positron abundance and typically higher injected activities allowed from a radiation safety perspective.

## Conclusion

In this study, we found, when using the SiPM-based Vision PET/CT for ^89^Zr immunoPET imaging and the EARL standard 1 compliant reconstruction settings, semiquantitative PET image parameters to remain reliable when using images obtained at reduced scan durations up to a factor of 2 compared to using the conventional PMT-based mCT PET/CT system.

Also, as SUV_max_ is highly affected by noise and reconstruction settings, and differs considerably in quantification of tumour lesions as well as healthy tissues between various PET/CT systems, we strongly recommend using the EARL standard 1 compliant reconstruction protocol and to report SUV_peak_ for reliable, comparable across systems, tumour lesion quantification in ^89^Zr PET/CT imaging.

## Supplementary Information

Below is the link to the electronic supplementary material.

Supplementary file1 (DOCX 128 KB)

## Data Availability

The datasets generated and analysed during the current study are not publicly available due to sensitive information, but could be made available in anonymous form from the corresponding author on reasonable request.
