# Supplementary material for: Optimisation of scan duration and image quality in oncological 89Zr immunoPET imaging using the Biograph Vision PET/CT
Source: Eur J Nucl Med Mol Imaging. 2023 Mar 22;50(8):2258–70. doi: 10.1007/s00259-023-06194-4 (PMC10250429; doi:10.1007/s00259-023-06194-4)
Supplement: Supplementary file 1 — Supplementary file1 (DOCX 128 KB) [file 259_2023_6194_MOESM1_ESM.docx]

**SUPPLEMENTAL DATA**

Visual Image Assessment Form

**Image code: ……………………………………….**

1. **Image noise is:**

Enormous Disturbing Acceptable Hardly there Not perceivable

1. **The lesion margin delineation:**

Cannot be confirmed Vague Acceptable Pretty clear Excellent

1. **Overall image quality is:**

Poor Suboptimal Acceptable Good Excellent

Supplemental Figure 1 – Image quality assessment form based on a five-point Likert scale used by two nuclear medicine physicians for visual assessment of image noise, tumour lesion margin demarcation, and overall image quality.


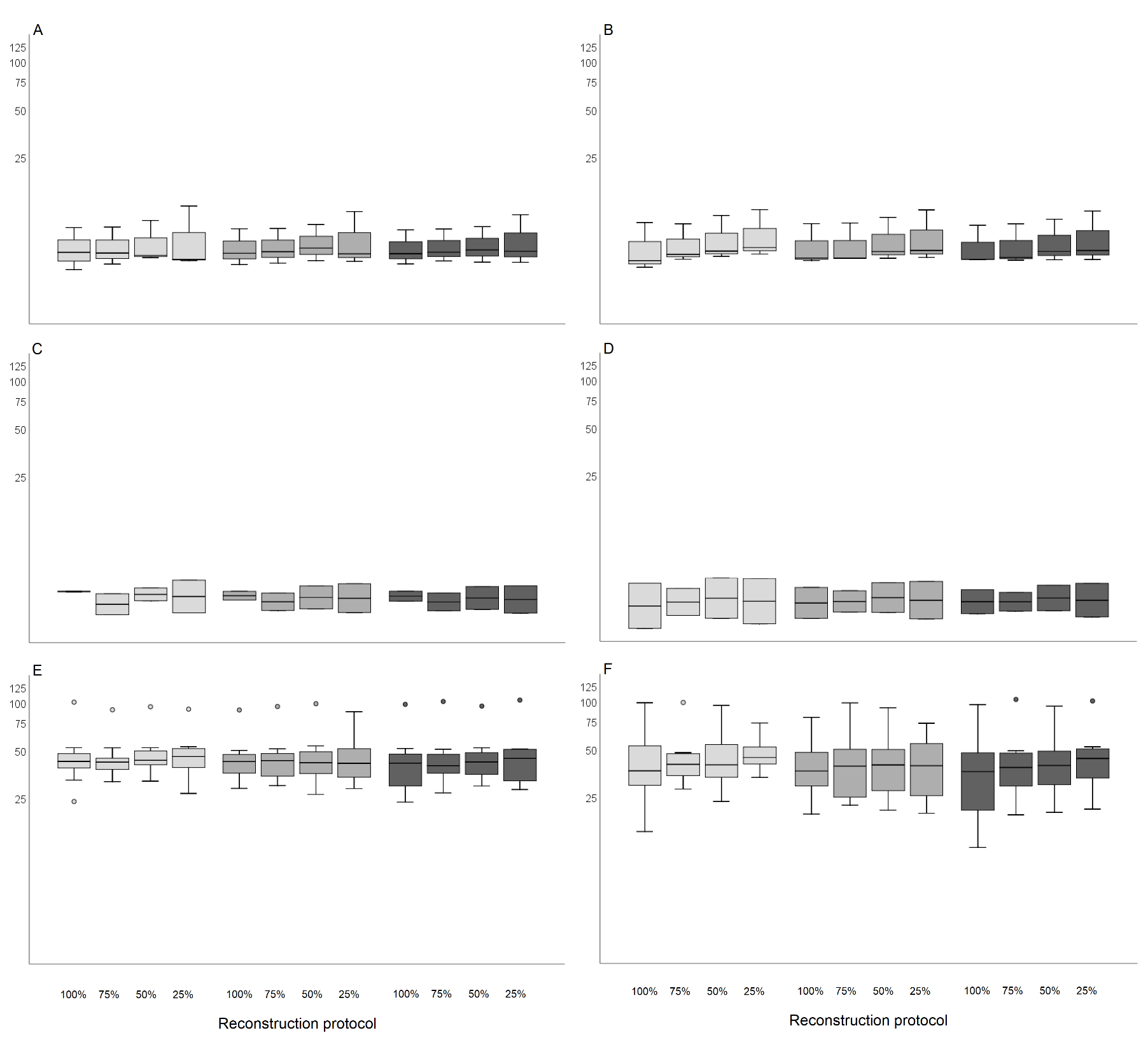


Supplemental Figure 2 – Vision only semi-quantitative healthy tissue comparison between scan durations. For all patients (n = 15), SUV_peak_ (left column) and SUV_mean_ (right column) of the spleen using three different ^89^Zr-labeled mAbs (tracer 1 for n = 3 (A and B), tracer 2 for n = 2 (C and D), and tracer 3 for n = 10 (E and F). PET/CT imaging was performed on the Vision system using the Clinical Vision reconstruction protocol (light grey), the EARL2 Vision reconstruction protocol (grey), and the EARL1 Vision reconstruction protocol (dark grey) are compared at different scan durations (100% to 25%, from left to right for each reconstruction protocol).
